# Accessory mitral valve tissue that caused a left ventricular outflow tract obstruction: a case report

**DOI:** 10.1186/s40981-019-0306-2

**Published:** 2019-12-30

**Authors:** Takashi Tennichi, Takumi Taniguchi

**Affiliations:** Department of Anesthesiology and Intensive Care Medicine, Kanazawa University, 13-1 Takara-machi, Kanazawa, Ishikawa 920-8641 Japan

**Keywords:** Accessory mitral valve tissue, Left ventricular outflow tract obstruction, Transthoracic echocardiography, Transesophageal echocardiography

## Abstract

**Background:**

Accessory mitral valve tissue (AMVT) is a rare congenital cardiac anomaly and is usually diagnosed in childhood. The diagnosis of AMVT in adulthood is extremely rare. We present a case report on an adult patient with AMVT that caused a left ventricular outflow tract (LVOT) obstruction.

**Case presentation:**

A 51-year-old man was diagnosed with AMVT via transesophageal echocardiography, which resulted in an LVOT occlusion (mean gradient 12 mmHg) during systole. Resection of the AMVT was performed under general anesthesia. The patient was hemodynamically stable throughout the surgery and post-operation. There was no abnormity of the mitral valves, including mitral regurgitation.

**Conclusions:**

Although a very rare malformation, particularly in adults, AMVT can cause LVOT obstruction. Examination of the mitral valve using transesophageal echocardiography is important to understand the severity of LVOT obstruction.

## Background

Accessory mitral valve tissue (AMVT) is a rare congenital cardiac anomaly. In some cases, it may lead to left ventricular outflow tract (LVOT) obstruction. Systolic ballooning of the AMVT into the outflow tract results in a mass effect and subaortic obstruction, causing symptoms such as exercise intolerance with dyspnea, chest pain, and syncope [1]. Approximately 70% of cases are diagnosed in childhood (including neonatal period), often based on signs or symptoms related to obstruction [2]. Therefore, diagnosis of this cardiac anomaly in adulthood is extremely rare [3–5]. We present a case report on an adult patient with AMVT that caused an LVOT obstruction.

## Case presentation

A 51-year-old man (height 174 cm, weight 78 kg) was diagnosed with a cardiac murmur in childhood. However, he did not have any further detailed cardiac examinations.

He had been suffering from chest pain since the age of 50. His chest pain was atypical with some non-specific characteristics. At this time, transthoracic echocardiography (TTE) was conducted, which showed a string-like abnormal structure in the LVOT. However, he did not follow up because he was asymptomatic. After a year, his chest pain recurred. TTE revealed the same structural abnormality again. Therefore, he underwent a detailed examination. Except for the cardiac murmur, he had no medical history and was not on any medications, and there was no family history of cardiac problems.

A physical examination revealed a Levine type III to-and-fro heart murmur in the aortic area. His blood pressure was 123/79 mmHg, pulse rate was 64 beats per minute (bpm), and his lungs were clear on auscultation. A chest X-ray showed a normal cardiac silhouette and both lungs were clear and expanded, with no infiltrates or pleural effusions. An electrocardiogram (ECG) showed non-specific changes and a normal sinus rhythm.

**Fig. 1:**
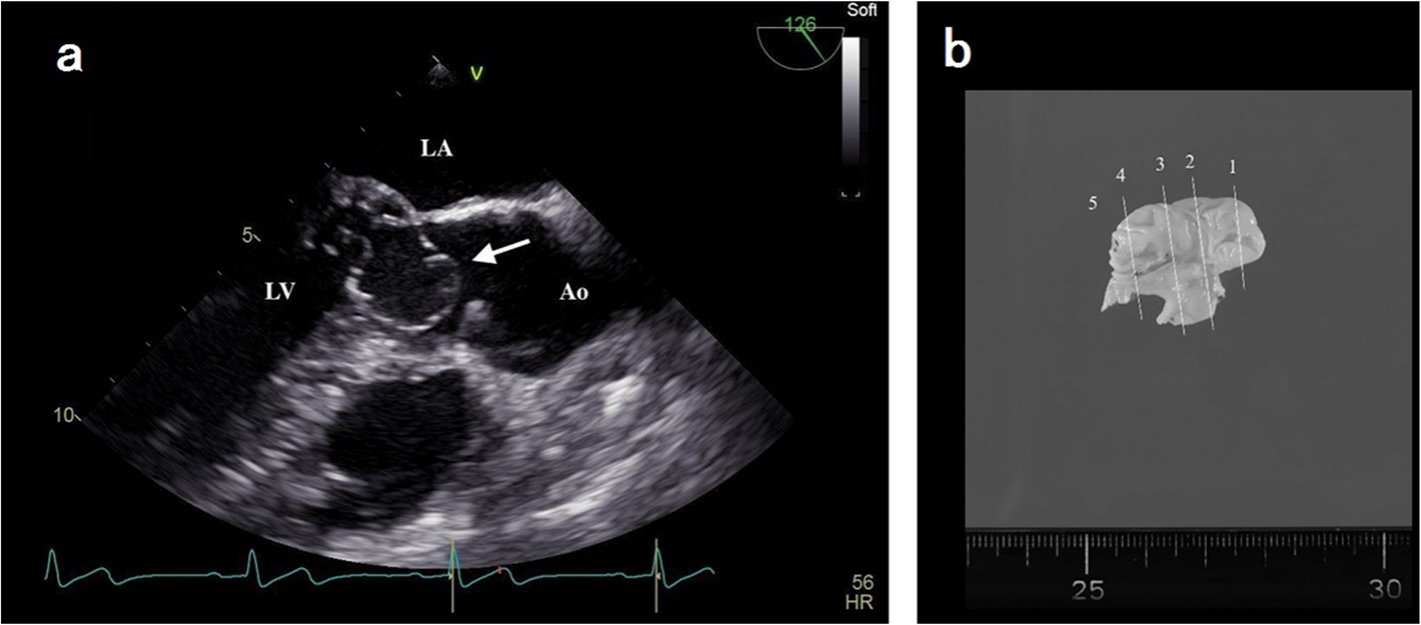
**a** Mid esophageal aortic valve long-axis view on transesophageal echocardiography. The white arrow shows the accessory mitral valve tissue (AMVT). Ao, aorta; LA, left atria; LV, left ventricle. **b** Resected AMVT

**Fig. 2:**
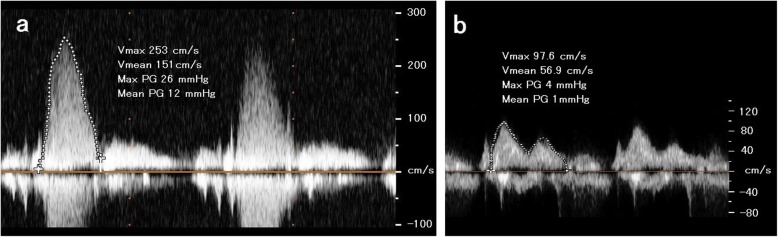
**a** Transaortic echocardiography pressure peak gradient, measured by continuous wave Doppler across the left ventricular outflow tract before surgery. **b** Transaortic echocardiography pressure peak gradient measured by continuous wave Doppler across the left ventricular outflow tract after surgery. Vmax, maximum flow velocity; Vmean, mean flow velocity; Max PG, maximum pressure gradient; Mean PG, mean pressure gradient

Transesophageal echocardiography (TEE) revealed an oval-like tissue with clean margins attached to the anterior leaflet of the mitral valve, causing an LVOT occlusion during systole (Fig. 1a). The maximum gradient pressure through the LVOT was measured at 26 mmHg with a mean gradient of 12 mmHg (Fig. 2a). The left ventricle wall motion was normal. The dimensions of the left ventricle during both systolic and diastolic phases were normal. No other cardiac anomalies were present. A diagnosis of AMVT was made based on the echocardiographic findings. Surgical treatment was recommended because of the presence of AMVT and the significant LVOT obstruction.

Surgery was scheduled for resection of the AMVT. In the operating room, after placing an arterial catheter in the right radial artery to continually measure the patient’s blood pressure, we induced general anesthesia by intravenous administration of midazolam 10 mg, fentanyl 500 μg, and rocuronium 70 mg. A central venous catheter and pulmonary arterial catheter were inserted via the right internal jugular vein. Anesthesia was maintained with oxygen, sevoflurane, and propofol. Bolus intravenous fentanyl infusion was administered as needed. After starting extracorporeal cardiopulmonary bypass (CPB), the AMVT was resected via the aortic valves (Fig. 1b). After resecting the AMVT, we began removing the CPB. We ensured the LVOT was no longer obstructed, but severe mitral regurgitation (MR) was observed on TEE. Therefore, a mitral valvuloplasty was conducted under CPB. MR ceased after mitral valvuloplasty, and subsequently, we stopped using CPB with dobutamine at 4 μg/kg/min and commenced biventricular pacing (90 bpm). The mean arterial blood pressure was maintained at 60–70 mmHg, the central venous pressure (CVP) at 10–15 mmHg, and the pulmonary arterial pressure (PAP) at 20–30 mmHg.

The total surgical duration was 313 mins, CPB duration was 108 mins, and the anesthesia duration was 414 mins.

**Fig. 3:**
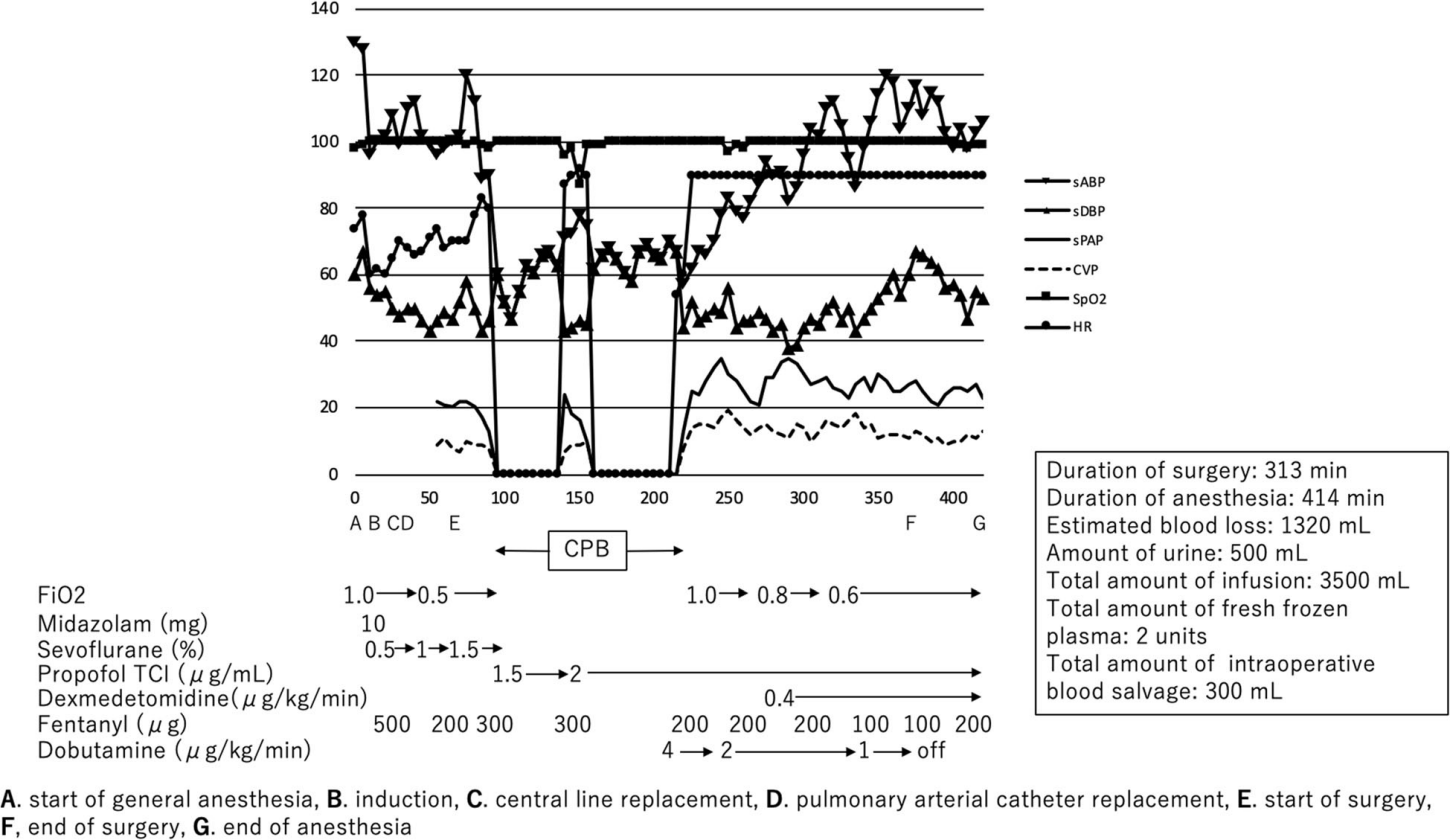
Intraoperative hemodynamic and respiratory changes during the surgery

The patient received a total of 3500 mL of lactated Ringer’s solution, 300 mL of intraoperative blood salvage, and 2 units of fresh frozen plasma during the procedure. His estimated blood loss was 1320 mL. The patient was hemodynamically stable throughout the surgery, with no abnormal findings on the ECG (Fig. 3). After the surgery, the patient was transported to the intensive care unit (ICU) without awakening or extubating.

The patient was extubated 6 h after being transported to the ICU. His hemodynamic state was stable with dobutamine at 0.6–1.3 μg/kg/min. There was no abnormity of the mitral valves including mitral regurgitation, and the maximum gradient pressure through the LVOT was measured at 4 mmHg with a mean gradient of 1 mmHg (Fig. 2b). Dobutamine was stopped on the second postoperative day. He responded well to treatment and was discharged 18 days after surgery.

## Discussion

In this case, the patient was diagnosed with AMVT causing an LVOT obstruction in adulthood, and anesthetic management after AMVT resection was performed without any complications.

AMVT is usually diagnosed in childhood because it is often associated with other congenital cardiac anomalies, and symptoms of LVOT obstruction often develop early on in life. Therefore, a case of AMVT diagnosed in adulthood is extremely rare [3–5]. In our case, the patient remained asymptomatic for many years and was first diagnosed with AMVT as an adult, using TTE and TEE.

Resection of AMVT is recommended if patients become symptomatic [4]. The current approach is to intervene if a patient has a significant LVOT gradient (a mean gradient of more than 25 mmHg) [6–8]. It is recommended that patients with an LVOT mean gradient under 25 mmHg are followed up at regular intervals [6, 7].

However, another case report stated that surgery should be performed as soon as possible on patients with AMVT, even if LVOT obstruction is not present, to prevent any future occlusions caused by the AMVT [9]. In the present case, surgery was performed despite the mean LVOT gradient of 12 mmHg, because the possibility of an embolism and further increases in the pressure gradient caused by the AMVT could not be ruled out.

During anesthetic management, it is important to maintain the pressure gradient of the LVOT until the patient is placed on CPB. A decrease in the ventricular preload and afterload presents a risk of exacerbating an LVOT obstruction. Therefore, a decrease in left ventricular volume, caused by vasodilatation while inducing anesthesia, should be avoided. In this case, the pressure gradient of the LVOT before surgery was 12 mmHg. However, we could not exclude the possibility of a temporary increase in the pressure gradient of the LVOT at the time the patient complained of chest pain. Therefore, we aimed to avoid excessive changes to hemodynamic stability, such as tachycardia and elevation of blood pressure during the induction of anesthesia, and changes to the preload and afterload. Furthermore, it was necessary to use TEE to carefully check the configuration of the mitral valve after resecting the AMVT. In this case, we identified MR using TEE and finished the surgery after mitral valvuloplasty.

Perioperative TEE is helpful for diagnosis confirmation and evaluation of the mitral valve after excision [9, 10]. TEE is also important in the evaluation of potential complications that may occur after excision and revision of cardiopulmonary bypass for repair and completion of the surgical procedure [11]. Therefore, anesthesiologists should be aware of how the mitral valve is functioning during surgery by using TEE, allowing the surgery to go as smoothly as possible in consultation with the surgeon.

In conclusion, despite being a rare cardiac malformation, especially in adults, a case of AMVT causing an LVOT obstruction was observed in a 51-year-old man. TEE examination of the mitral valve is important to understand the severity of LVOT obstruction before surgery. The results of TEE should be considered while planning intraoperative anesthetic management.

## Data Availability

Not applicable

## Abbreviations

AMVT: Accessory mitral valve tissue
bpm: Beats per minute
CPB: Cardiopulmonary bypass
CVP: Central venous pressure
ECG: Electrocardiogram
ICU: Intensive care unit
LVOT: Left ventricular outflow tract
MR: Mitral regurgitation
PAP: Pulmonary arterial pressure
TEE: Transesophageal echocardiography
TTE: Transthoracic echocardiography
